# Human and Animal Vaccination Delivery to Remote Nomadic Families, Chad 

**DOI:** 10.3201/eid1303.060391

**Published:** 2007-03

**Authors:** Esther Schelling, Mahamat Bechir, Mahamat Abdoulaye Ahmed, Kaspar Wyss, Thomas F. Randolph, Jakob Zinsstag

**Affiliations:** * Swiss Tropical Institute, Basel, Switzerland; † Centre de Support en Santé Internationale-Institut Tropical Suisse, N’Djaména, Chad; ‡ International Livestock Research Institute, Nairobi, Kenya; 1 Current affiliation: International Livestock Research Institute, Nairobi, Kenya

**Keywords:** Vaccination delivery, children, women, livestock, remote rural zones, measles, polio, anthrax, synopsis

## Abstract

Vaccination services for people and livestock often fail to achieve sufficient coverages in Africa’s remote rural settings because of financial, logistic, and service delivery constraints. In Chad from 2000 through 2005, we demonstrated the feasibility of combining vaccination programs for nomadic pastoralists and their livestock. Sharing of transport logistics and equipment between physicians and veterinarians reduced total costs. Joint delivery of human and animal health services is adapted to and highly valued by hard-to-reach pastoralists. In intervention zones, for the first time ≈10% of nomadic children (>1–11 months of age) were fully immunized annually and more children and women were vaccinated per day during joint vaccination rounds than during vaccination of persons only and not their livestock (130 vs. 100, p<0.001). By optimizing use of limited logistical and human resources, public health and veterinary services both become more effective, especially at the district level.

## Abstract 2

Vaccinating nomadic pastoralists and their livestock at the same time reduces delivery costs.

Vaccinating persons and livestock in hard-to-reach rural communities remains a serious challenge and calls for innovative, specially designed strategies. In Africa, the ability of human and veterinary health systems to deliver services is constrained by decreasing public-sector budgets; loss of confidence due to unmet demand; a severe shortage of human resources, especially qualified personnel (*1*); inadequate infrastructure and equipment; and weak monitoring and information systems (*2*). Most governments rely heavily on complementary donor funding from bilateral and multilateral partners.

One of the 4 strategies of the World Health Organization (WHO) and the United Nations International Children’s Emergency Fund to achieve their vision of equitable, sustainable, and high-coverage immunization among children and women by the year 2015 is the provision of vaccination services linked to other health interventions. To achieve common health goals, the cross-over benefits of integrating other interventions in public health such as distribution of vitamin A, selling of insecticide-treated mosquito nets, and deworming and malaria treatments of children are increasingly being acknowledged (*3*). Initial evaluations on cost-effectiveness of combined approaches (*4*) provide the needed data for more efficient planning.

For children and for women of childbearing age, immunization provided through the National Expanded Program on Immunization is considered one of the most cost-effective public health interventions and society’s best healthcare investment, especially in developing countries (*5*,*6*). National and global efforts aim to substantially increase vaccination coverage in general, particularly for those who have so far not benefited. Global coverage of fully immunized children is 78%, but in the sub-Saharan Africa (WHO African region) it is only 67% (*7*). A setback for the global polio eradication program was direct and indirect spread of wild polio virus type 1 from Nigeria to 11 African countries from 2002 through 2004. Affected countries that did not experience sustained transmission of this virus type had an average polio vaccination coverage of 83%, in contrast to the others that did have sustained transmission and had a much lower mean coverage of 52% (*8*). The virus spread directly into neighboring Chad and was further disseminated from there. With vaccination coverage <50% (*7*) and a large disparity between urban and rural vaccination coverage (20% vs. 9% for fully immunized children in 1997 [*9*]), Chadian public health professionals expressed their need for strategies to better reach the remote pastoral communities in the border regions to establish a “cordon sanitaire.”

The mobility and dispersion of mobile livestock owners in semiarid Africa and elsewhere lead to difficulties in reaching them with preventive and curative treatments as well as with information and education. Outreach human vaccination services rarely exist for rural communities living far (>15 km) from the nearest health facility, which in Chad accounts for 40% of the rural population (*9*). Especially difficult is providing health services to nomadic groups, which also represent substantial numbers. Although now out of date, the most recent Chadian census of 1993 registered 9.3% (83,500) nomads in a rural population of 900,000 in the Chari-Baguirmi and Kanem Districts of Chad (*10*); these figures are probably underestimated because during the census period (April), several nomadic groups would have been seasonally located across the border in neighboring countries. Evaluating effective coverage of such groups is particularly problematic; the records of rural health centers do not distinguish between the ways of life (settled or mobile) of vaccinated persons nor do health centers have appropriate stratified population numbers (numbers have simply been extrapolated annually since 1993) to permit calculating coverages in settled and mobile communities.

Strategies of vaccinating mobile pastoralists on market days have been tested; however, children and women tend to stay in their camps, resulting in very low participation (*11*). Mobile services cost more than stationary facility services (*12*), but preventive interventions in remote zones are hardly possible if not mobile. In 2000, the prevalence of fully immunized nomadic children and women in Chadian Chari-Baguirmi and Kanem was zero (*13*); in the same nomadic camps, however, livestock were compulsorily vaccinated by mobile veterinary teams.

Livestock are part of the livelihood of <70% of the world’s rural poor (*14*). Vaccination of livestock is a central tool for control and elimination of contagious livestock diseases. Elimination of foot and mouth disease with vaccination strategies, for example, can provide a country or a region access to export markets and dramatically increase a country’s benefits from veterinary intervention (*15*). Some veterinary vaccines prevent not only important production losses of livestock but also human disease (e.g., rabies of dogs and anthrax of ruminants). Anthrax spores remain dormant for years in soil until they infect other animals. Control of anthrax in a disease-endemic setting such as the Sahel includes educating persons about how handling and consuming infected livestock products influence risk of infection, meat inspection, and particularly yearly vaccination of livestock. In Africa during the 20th century, Rinderpest virus persisted between pandemics in nomadic or seminomadic pastoralists’ herds and in wildlife (*16*), from which it could spread anew to neighboring zones, as occurred in Ethiopia in the 1990s (*17*). Although a thermostable, efficacious, and safe vaccine exists, inaccessibility of cattle populations (because of remote locations, political crises, and insecurity) postponed the successful elimination of Rinderpest virus in livestock by the Pan-African Rinderpest Campaign.

Professionals from WHO and the Food and Agriculture Organization of the United Nations and others have proposed sharing of resources by public health and veterinary services to deliver health interventions at lower costs, thereby allowing for economies of scale (*18*,*19*). Few examples of joint implementation exist and evaluations of these experiences are rare (*20*). We review field research on joint human and animal vaccination campaigns among nomadic pastoralists of Chad and advocate for intersectoral approaches for service delivery in similar remote rural settings.

## Joint Delivery of Public Health and Veterinary Services for Nomadic Pastoralists

The Chadian Ministries of Health and of Livestock Production (hosting the veterinary services), together with the nomadic communities, recommended testing the feasibility of joint human and livestock vaccination campaigns. The goals of these joint campaigns were to make best use of visits by professionals in nomadic communities, reduce costs of services by sharing of infrastructure, and increase vaccination coverage. 

### Organization of Campaigns and Vaccinated Children and Women and Livestock

The Swiss Tropical Institute supported the implementation of several joint campaigns. From 2000 through 2001 and from 2003 through 2005, 14 vaccination campaigns for nomadic children, women, and the camps’ livestock were conducted among the 3 principal nomadic ethnic groups (Fulani, Arabs, and Dazagada) in the Chari-Baguirmi and Kanem of western Chad. Five campaigns were conducted in the zone of Gredaya and 3 campaigns each in Dourbali, AmDobak, and Chaddra (Table 1). Each campaign was composed of 3 vaccination rounds to ensure a complete course of vaccination of children in 1 year. The capacity of existing mobile veterinary teams was extended for simultaneous vaccination of people and animals during at least 1 round for 10 of the 14 campaigns (Table 1).

**Table 1:** Overview of 14 vaccination campaigns among mobile pastoralists in 4 zones of Chari-Baguirmi and Kanem Prefectures, Chad*

Zone	Campaign no.	Beginning–end of campaign	No. vaccination days	Vaccination contacts		% Dropout	
				Children and women	Livestock	Children	Women
Gredaya†	1	Jul 7, 2000–Feb 11, 2001	46	11,731	31,721‡	56	21
Chaddra†	2	Jan 6–Apr 21, 2001	26	1,961	2,182	68	7
Gredaya†	3	Jun 21–Nov 11, 2001	37	3,855	22,760	–	–
AmDobak†	4	Aug 3–Dec 5, 2001	34	3,079	0	59	42
Gredaya§	5	Apr 2–Jul 1, 2003	46	5,595	16,138	45	19
Dourbali§	6	Mar 8–Jun 7, 2003	47	6,715	887	72	41
Chaddra	7	Sep 27, 2003–Jan 3, 2004	43	3,031	215	–	–
AmDobak	8	Oct 19–Dec 6, 2003	29	3,049¶	0	–	–
Gredaya§	9	Mar 5–Jul 5, 2004	54	6,522	24,514	68	33
Dourbali§	10	Apr 29–Jul 25, 2004	54	3,370	5,104	89	57
Gredaya	11	Jan 10–Mar 30, 2005	42	3,883	13,217	–	–
Dourbali	12	May 18–Aug 17, 2005	41	3,477	32,517	–	–
AmDobak	13	Oct 23–Dec 7, 2005	21	2,705¶	0	–	–
Chaddra	14	Oct 16, 2005–Jan 9, 2006	31	1,886	0	–	–

*Dropout rates were calculated for the first campaign in each zone (vaccination in a naive population) and for 2003 and 2004 after omission of persons with previous vaccinations and not considering those entering the campaign during the second and third rounds. –, not applicable. †Costing study. ‡Two rounds with veterinarians. §Estimation of achieved vaccination coverage during campaign. ¶Only 2 human vaccination rounds carried out.

The joint campaigns were organized in consultation with local health and veterinary personnel to avoid duplication of efforts and to make use of all existing personnel and infrastructure (cold chain and transportation means). Pictograms and short movies with health and veterinary messages were shown to and discussed with nomadic community members before vaccinations were given by trained community-based facilitators. The National Expanded Program on Immunization provided human vaccines and consumables such as syringes through the regional health administration, assessed continuously the number of vaccinated persons, and was involved in evaluation of the achieved vaccination coverage (*20*).

Veterinarians vaccinated 149,255 livestock against anthrax, pasteurellosis, blackleg, and contagious bovine pleuropneumonia. After 3 visits from the vaccination team, 4,653 children <5 years of age were fully immunized against diphtheria, whooping cough (pertussis), and tetanus (DPT) and against polio; 7,703 women received at least 2 doses of tetanus vaccine (TT2+). The average dropout rate within a given campaign was 64% for children <5 years from the first to third vaccination for polio and DPT within a given campaign and 32% for women from the first to second dose of tetanus vaccine (Table 1). Dropping out was caused rarely by refusal of revaccination, more often by high mobility of nomadic families. Achieving smaller dropout rates remains a critical need, although DPT and polio vaccination can be continued in subsequent years, and 1 vaccination contact is already useful for immunizing children against measles and yellow fever.

### Assessment of Costs

An assessment of costs for the vaccination years 2000 and 2001 (*21*) used unit costs based on detailed data (e.g., replacement costs, maintenance, and insurance costs) and local prices. Purchase prices of human vaccines obtained from the Pan American Health Organization (*22*) were adjusted to include supply, transport, and storage costs and a wastage rate of 10%. The purchase costs of livestock vaccines at the national veterinary laboratory were adjusted for a wastage rate of 5%. Private costs incurred by households to participate in the vaccination campaign were not included in this study. Results are given in Euros (1€ = 656 Francs de la Communauté Financière d’Afrique, September 2006). To allocate the costs of resources used jointly by the 2 sectors, the costs of the vehicle(s), fuel, and guides have been distributed proportionally to the number of personnel present during joint campaigns. The costs of the cold chain, program coordination and administration, information campaigns, and the vehicle and fuel during preparative missions have been distributed according to the number of vaccination rounds by sector. Cold-chain costs were shared with the livestock sector only when veterinarians used thermosensitive contagious bovine pleuropneumonia vaccines. The proportion of public health costs saved by cost-sharing was calculated on the basis of independent public health sector campaigns (*23*).

Most costs of vaccination campaigns for both sectors were the variable and fixed costs of supplying vaccines and vaccine-related costs such as syringes and needles (46% for public health and 53% for veterinary sector) (Table 2). Livestock owners were charged €0.15 per veterinary vaccine dose administered. This covered the costs in Gredaya but not in AmDobak/Chaddra (Table 3). In Gredaya, a higher proportion of personnel and transportation could be shared than in Chaddra/AmDobak because of more joint campaigns (3 of 6 rounds) than in Chaddra/AmDobak (1 of 6 rounds). Of total costs, the proportion shared across the veterinary and public health sectors in the 2 districts was 6.7% and 2.8%, respectively. These proportions were considerably higher when vaccine costs that cannot be shared between sectors were excluded: 15.1% and 4.1%, respectively (Table 2). Costs per fully immunized child and tetanus-vaccinated (TT2+) woman were €11.9 and €6.8, respectively, in Gredaya, but comparatively higher in Chaddra/AmDobak at €30.3 and €18.7, respectively (Table 2). Loss to follow-up (dropout rates) was crucial for the calculated costs per outcome because each incompletely immunized child and woman added to the total costs but did not appear in the denominator. The cost-effectiveness and running costs are currently being evaluated by combining vaccination coverage and cost data of both sectors and including household costs.

**Table 2:** Variable and fixed costs of vaccinations in the veterinary and public health sectors in Gredaya and AmDobak/Chaddra, Chad*

Cost	Veterinary sector		Public health sector			
	Gredaya, Euros (% Fixed)	Chaddra/AmDobak, Euros (% Fixed)	Gredaya		Chaddra/AmDobak	
			Euros (% Fixed)	% Shared	Euros (% Fixed)	% Shared
Personnel/administration	2,559 (0)	475 (0)	3,627 (0)	10.6	3,376 (0)	2.7
Transportation	2,835 (80)	345 (75)	4,004 (82)	19.3	3,797 (79)	3.3
Cold chain	62 (36)	45 (56)	1,185 (37)	6.2	531 (36)	10.1
Vaccines and vaccines-related	7,541 (29)	214 (21)	12,146 (12)	0	4,072 (12)	0
Other (buildings, supplies)	480 (95)	152 (100)	938 (98)	25.4	938 (98)	9.1
Total costs	13,476	1,231	21,900	6.7	12,712	2.8
Total costs without vaccines	5,935	1,025	9,754	15.1	8641	4.1

*In Gredaya, 3 vaccination rounds were conducted jointly between veterinarians and public health professionals and another 3 rounds were conducted by the public health sector alone to fully immunize children, whereas in Chaddra/AmDobak, only 1 of 6 rounds was conducted jointly with the veterinarians. The cost-sharing scheme and the proportion of reduced costs due to the joint approach are described in the text.

**Table 3:** Cost per vaccinated livestock and cost and marginal cost per fully immunized child and woman in Gredaya and Chaddra/AmDobak, Chad*

Cost per outcome	Veterinary sector		Public health sector			
	Gredaya, Livestock	Chaddra/AmDobak, Livestock	Gredaya		Chaddra/AmDobak	
			FIC	TT2+	FIC	TT2+
Total no. vaccinated	54,185	2,182	1,697	1,679	405	488
Cost per vaccine dose administered	0.11	0.56	0.6	2.0	1.1	5.6
Cost per vaccinated livestock† or fully immunized child or woman	0.25	0.56	11.9	6.8	30.3	18.7
Marginal cost per FIC/TT2+‡			8.7	4.6	13.6	5.0

*FIC, fully immunized child; TT2+, woman with at least 2 antitetanus vaccinations.  †Cost per vaccinated animal: either vaccination against anthrax, blackleg and pasteurellosis, or against contagious bovine pleuropneumonia at 1 encounter. ‡Marginal cost per FIC and per TT2+ at a capacity limit of 200 vaccinated children and 100 women per day.

### Achieved Vaccination Coverage

Sizes of the pastoralist communities were largely unknown. To calculate the proportion of children and women reached in Gredaya and Dourbali in 2003 and 2004, initial population sizes have been estimated by the mark-recapture method. Vaccination cards were used as marks; then during transect studies 1 year after the vaccination, the vaccination status of randomly selected persons was recorded. A Bayesian model enabled us to combine different population estimates of nomadic groups and recapture probabilities to obtain estimates of population dynamics and population sizes at time of vaccination. Estimated coverages of fully immunized children 0–11 months of age (DPT3/polio3) were 8% in 2003 and 14% in 2004 in Gredaya, and 8% in 2003 and 7% in 2004 in Dourbali. Proportions of women who received TT2+ were 16% in 2003 and 36% in 2004 in Gredaya, and 13% in 2003 and 11% in 2004 in Dourbali. No cumulative coverage could be calculated because the total population sizes in the vaccination zones varied between years. A trend toward saturation of fully immunized children (12–36 months of age) and women could not be shown. Further complicating evaluation, new nomadic families enter the vaccination zones because of flexible routes and schedules. This dynamic is reflected in high estimated emigration rates (on average involving 70% of the total population).

### Perception of Campaigns

Pastoralist communities highly value the combined approach that considers the health of their family members and of their livestock (Figure) (*20*). In addition to anecdotal positive feedback from pastoralists and health and veterinary personnel, our data collected during the 14 campaigns showed a higher mean of 131 (95% confidence interval [CI] 115–148) persons vaccinated per day for 176 combined vaccination days compared with 100 (95% CI 94–106) for 377 days when only persons, and not livestock, were vaccinated (p<0.001 by Poisson regression with random effect on zone and adjusted to number of vaccination rounds). Pastoralists no longer refused vaccination of their livestock as had sometimes been the case before veterinarians were accompanied by medical personnel. A key statement repeatedly made by nomadic parents was, “Measles and whooping cough have disappeared among nomads, although it remains at the market-sites we visit. And when we attend markets, we no longer contaminate our camps with these diseases.” 

**Figure:**
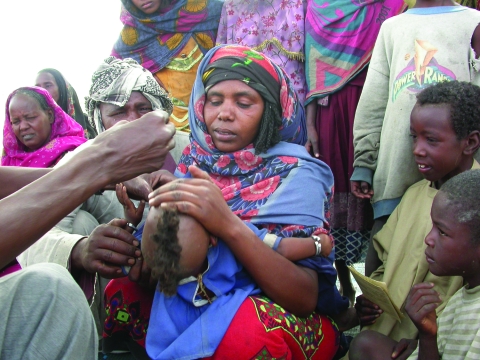
Polio vaccination of a nomadic child in Chad. While children and woman in the camp received vaccinations by public health workers, the livestock in the camp received vaccinations by veterinarians. Source: Project Santé des Nomades au Tchad.

Typically, the initial contact between nomadic families and health personnel is established during the vaccination program (*24*). The nomadic pastoralists perceive the high-quality services that are offered at the health center and start to trust the health providers. The public health sector was thus able to use the campaigns as a gateway to the pastoralists. Increasingly, nomadic parents are visiting health centers with their children to seek vaccination services. Based on the positive outcomes of these pilot campaigns, Chadian public health and veterinary officials are currently planning a common policy for child and livestock vaccination in pastoralist populations. Going to scale at district and national levels with combined public health and veterinary campaigns is sought in concert with other ministries such as education. This may become a model for other governments who face similar difficulties in reaching remote livestock keepers because of communities reluctant to comply with public or private officials or insufficient infrastructure and resources.

## Identifying Synergies in Face of Public Sector Financial Constraints

Privatization of veterinary services was initiated in many parts of Africa and Asia as part of a broader effort to improve delivery of animal healthcare in the face of decreasing governmental expenditure and poor public sector performance (*25*). Numerous incentive schemes were designed to stimulate the privatization process. Subcontracted veterinarians can be effective in the implementation of vaccination campaigns, given that the government subsidizes work in more remote zones. Our analysis on vaccination costs of private veterinarians showed that even with subsidies, income margins of private veterinarians in rural zones for vaccination services (and vaccination being their main activity) were narrow, and most private veterinarians who had started business in rural zones had since withdrawn. Community-based animal health service projects partly fill the gap in chronically underserved rural areas (*26*). However, to efficiently reach remote zones for mass vaccination campaigns, vehicles are needed to maintain the cold chain that cannot be achieved with other transportation means such as horses. In some instances, there is a lack of independent and regular quality control of veterinary vaccine production facilities in low-income countries (*27*). Insufficient vaccination coverage was commonly believed to be responsible for anthrax outbreaks in Chad. However, a survey showed that most pastoralists reported that vaccines were no longer efficacious. Subsequent, quality control of the anthrax vaccine confirmed the concerns of the livestock owners (*23*).

To ensure effective surveillance and vaccination coverage, which were once provided by the government, veterinary privatization policy is currently under review by the World Organization for Animal Health, the Food and Agriculture Organization of the United Nations, and the African Union (Regional Conference in N’Djaména, February 13–15, 2006). New policies should be judged on whether they adequately support the rural poor in continuing livestock-based livelihoods (*2*,*28*), which in arid and semiarid zones are often the only way to productively use the natural resource base.

Exploiting synergies for interventions and information dissemination becomes more and more important. Within countries, regular intersectoral exchange of disease occurrence information adds to better preparedness in both sectors, but such exchange of information does not exist in all countries. The more recently evaluated institutional collaborations between public health and veterinary services seek to identify appropriate control strategies for diseases where medical and veterinary cooperation for control is becoming a single continuum. Transsectoral economic analyses of livestock disease control may demonstrate increased profitability and contribute to advocacy for improved control of zoonoses in developing countries (*29*).

## Conclusions

Sustained vaccination programs are essential tools for both the public health and veterinary sectors. Combined human and livestock vaccination reduces operational costs of interventions requiring costly transportation and is adapted to livestock holders who highly value the approach that considers the health both of the family and of the animals that contribute importantly to their livelihood. In Chad, a common policy agreement between the 2 sectors on cooperation in rural zones should define a cost-sharing scheme. By optimizing the use of limited logistical and human resources, public health and veterinary services will be strengthened, especially at the district level, and, in turn, will be more prepared and operational in responding to endemic and epidemic diseases.

## References

[1] Wyss K, Moto DM, Callewaert B. Constraints to scaling-up health related interventions: the case of Chad, Central Africa. J Int Dev. 2003;15:87–100. 10.1002/jid.967

[2] Cheneau Y, El Idrissi AH, Ward D. An assessment of the strengths and weaknesses of current veterinary systems in the developing world. Rev Sci Tech. 2004;23:351–9. 15200109 10.20506/rst.23.1.1489

[3] World Health Organization, United Nations International Children’s Emergency Fund. Global immunization vision and strategy 2006–2015. Geneva: The Organization. WHO/IVB/05.05; 2005.

[4] Grabowsky M, Nobiya T, Ahun M, Donna R, Lengor M, Zimmerman D, Distributing insecticide-treated bednets during measles vaccination: a low-cost means of achieving high and equitable coverage. Bull World Health Organ. 2005;83:195–201. 15798843 PMC2624203

[5] Jamison DT, Mosley WH, Measham AR, Bobadilla JL. Disease control: priorities in developing countries. New York: Oxford University Press; 1993.

[6] Chabot I, Goetghebeur MM, Gregoire JP. The societal value of universal childhood vaccination. Vaccine. 2004;22:1992–2005. 10.1016/j.vaccine.2003.10.027 15121312

[7] World Health Organization. Immunization vaccines and biologicals. WHO vaccine-preventable diseases: monitoring system. 2005 global summary. Geneva: The Organization; 2005.

[8] Centers for Disease Control and Prevention. Resurgence of wild poliovirus type 1 transmission and consequences of importation—21 countries, 2002–2005. MMWR Morb Mortal Wkly Rep. 2006;55:145–50. 16484977

[9] Ouagadjio B, Nodjimadji K, Ngoniri JN, Ngakoutou N, Ignégongba K. Tokindang JS, et al. Enquête démographique et de santé, Tchad 1996–1997. Calverton (MD): Bureau Central du Recensement et Macro International Inc.; 1998.

[10] Ministère du Plan et de la Coopération (MPC). Ministère de l’Intérieur et de la Sécurité. Recensement général de la population et de l’habitat 1993. N’Djaména (Chad): Le Ministère; 1995.

[11] Imperato PJ. The use of markets as vaccination sites in the Mali Republic. J Trop Med Hyg. 1969;72:8–13. 5773812

[12] Brenzel L, Claquin P. Immunization programs and their costs. Soc Sci Med. 1994;39:527–36. 10.1016/0277-9536(94)90095-7 7973852

[13] Daoud S, Yam A, Daugla DM, Schelling E, Diguimbaye C, Bidjeh K, Couverture vaccinale et prévalence des affectations courantes chez les nomades du Chari-Baguirmi et du Kanem au Tchad. Sempervira. 2000;8:37–43.

[14] Livestock in poverty-focused development. Somerset (UK): Livestock in Development; 1999.

[15] Randolph TF, Perry BD, Benigno CC, Santos IJ, Agbayani AL, Coleman P, The economic impact of foot and mouth disease control and eradication in the Philippines. Rev Sci Tech. 2002;21:645–61. 12523704 10.20506/rst.21.3.1355

[16] Rossiter PB. Rinderpest. In: Coetzer JA, Tustin RC, editors. Infectious diseases of livestock. Cape Town (South Africa): Oxford University Press Southern Africa; 2004. p. 629–59.

[17] Roeder PL. The animal story. BMJ. 2005;331:1262–4. 10.1136/bmj.331.7527.1262 16308392 PMC1289332

[18] Majok AA, Schwabe CW. Development among Africa’s migratory pastoralists. 1st ed. Westport (CT): Greenwood Publishing Group; 1996.

[19] Ward DE, Ruppaner R, Marchot PJ, Hansen JW. One medicine: practical application for non-sedentary pastoral populations. Nomad People. 1993;32:55–63.

[20] Schelling E, Wyss K, Bechir M, Moto DD, Zinsstag J. Synergy between public health and veterinary services to deliver human and animal health interventions in rural low income settings. BMJ. 2005;331:1264–7. 10.1136/bmj.331.7527.1264 16308393 PMC1289333

[21] Bechir M, Schelling E, Wyss K, Daugla DM, Daoud S, Tanner M, An innovative approach combining human and animal vaccination campaigns in nomadic settings of Chad: experiences and costs. Med Trop (Mars). 2004;64:497–502. 15771021

[22] Pan American Health Organization. PAHO’s revolving fund vaccine prizes for 2000. EPI newsletter: expanded program on immunization in the Americas. Vol 22. 2000; p.8.

[23] Schelling E. Human and animal health in nomadic pastoralist communities of Chad: zoonoses, morbidity and health services. 2002. Basel: University of Basel. [cited 2006 Dec 28]. Available from http://pages.unibas.ch/diss/2002/DissB_6478.htm

[24] Schelling E, Daoud S, Daugla DM, Diallo P, Tanner M, Zinsstag J. Morbidity and nutrition patterns of three nomadic pastoralist communities of Chad. Acta Trop. 2005;95:16–25. 10.1016/j.actatropica.2005.03.006 15866506

[25] Leonard DK. Africa’s changing markets for health and veterinary services. The new institutional issues. London: MacMillan Press Ltd; 2000.

[26] Woodford JD. Synergies between veterinarians and para-professionals in the public and private sectors: organisational and institutional relationships that facilitate the process of privatising animal health services in developing countries. Rev Sci Tech. 2004;23:115–35. 15200091 10.20506/rst.23.1.1472

[27] Thiaucourt F, van der Lugt JJ, Provost A. Contagious bovine pleuropneumonia. In: Coetzer JA, Tustin RC, editors. Infectious diseases of livestock. Cape Town (South Africa): Oxford University Press Southern Africa; 2004. p.2045–57.

[28] McLeod A, Wilsmore A. The delivery of animal health services to the poor: A review. Appendix 11. In: Perry BD, Randolph TF, McDermott JJ, Sones KR, Thornton PK, editors. Investing in animal health research to alleviate poverty. Nairobi (Kenya): International Livestock Research Institute; 2002. p.1–24.

[29] Zinsstag J, Schelling E, Wyss K, Mahamat MB. Potential of cooperation between human and animal health to strengthen health systems. Lancet. 2005;366:2142–5. 10.1016/S0140-6736(05)67731-8 16360795

